# Association of infant Rib and Alp1 surface protein *N*-terminal domain immunoglobulin G and invasive Group B Streptococcal disease in young infants

**DOI:** 10.1016/j.vaccine.2023.01.071

**Published:** 2023-03-03

**Authors:** Ziyaad Dangor, Gaurav Kwatra, Andrzej Pawlowski, Per B. Fisher, Alane Izu, Sanjay G. Lala, Bengt Johansson-Lindbom, Shabir A. Madhi

**Affiliations:** a South African Medical Research Council: Vaccines and Infectious Diseases Analytics Unit, University of the Witwatersrand, South Africa; b African Leadership in Vaccinology Expertise University of the Witwatersrand, South Africa; c Department of Paediatrics & Child Health, Faculty of Health Sciences, University of the Witwatersrand, South Africa; d Department of Clinical Microbiology, Christian Medical College, Vellore, India; e Immunology Section, BMC D14, Lund University, Lund, Sweden; f MinervaX ApS, DK-2200 Copenhagen N, Denmark

**Keywords:** GBS vaccine, Alp family proteins, Correlate, Immune responses

## Abstract

**Background:**

Vaccine development for Group B Streptococcus (GBS), a common cause of invasive disease in early-infancy and adverse pregnancy outcomes, include exploring widely-expressed GBS surface proteins as vaccine epitopes. We investigated the association between natural infant serum IgG against the RibN and Alp1N domains and risk of invasive GBS disease caused by isolates expressing these proteins.

**Methods:**

We analyzed maternal and infant serum samples from GBS disease cases and infants born to GBS-colonized women controls. Bayesian modelling was used to calculate the GBS homotypic IgG concentration associated with risk reduction of invasive disease in the infant.

**Results:**

PCR-based typing of 85 GBS invasive isolates showed 46 and 24 possessing the gene for Rib and Alp1, respectively. These were matched to 46 and 36 infant controls whose mothers were colonized with GBS expressing Rib and Alp1, respectively. RibN IgG geometric mean concentrations (GMC) were lower in cases than controls among infants (0.01; 95 %CI: 0.01–0.02 vs 0.04; 95 %CI: 0.03–0.06; p < 0.001), no significant difference was found between maternal RibN IgG GMC in cases compared to controls. Alp1N IgG GMC was also lower in infant cases (0.02; 95 %CI: 0.01–0.03) than controls (0.05; 95 %CI: 0.04–0.07; p < 0.001); albeit not so in mothers. An infant IgG threshold ≥ 0.428 and ≥ 0.112 µg/mL was associated with 90 % risk reduction of invasive GBS disease due to Rib and Alp1 expressing strains, respectively.

**Discussion:**

Lower serum RibN and Alp1N IgG GMC were evident in infants with invasive GBS disease compared with controls born to women colonized with GBS expressing the homotypic protein. These data support the evaluation of Alp family proteins as potential vaccine candidates against invasive GBS disease.

## Highlights

• Infant RibN IgG ≥ 0.428 µg/mL was associated with 90 % risk reduction of GBS disease.
• Infant Alp1N IgG ≥ 0.112 µg/mL was associated with 90 % risk reduction of GBS disease.
• These protein antibody thresholds are correlates of protection against GBS disease.
• Such data could contribute to the pathway of licensure of a maternal GBS vaccine.

## 1 Introduction

Invasive Group B Streptococcus (GBS) disease is a common cause of sepsis and meningitis in young infants, and may cause stillbirth [1], [2]. Preventative strategies, using intrapartum antibiotic prophylaxis in pregnant women has reduced the burden of early-onset disease (EOD) but not late-onset GBS disease (LOD) [3]. Maternal vaccination to protect the mother, fetus and young infants against GBS disease is being explored as alternate or complementary strategy against GBS disease [4]. A hexavalent conjugated capsular polysaccharide vaccines has reached phase–II evaluation and would cover about 98 % of the serotypes causing disease globally [5]. In addition, other vaccine candidates have been identified [6]: vaccines containing widely expressed surface proteins, including the *N*-terminal domains of members of the Alpha-like protein (Alp) family, have completed Phase I evaluation [7], [8] and are now undergoing Phase II evaluation (NCT05154578, NCT04596878). The Rib, Alpha C and Alp 1–4 proteins are the six members of the Alp family of GBS surface proteins [9], [10], [11]. Alp family proteins are found on most (99,7%) GBS isolates irrespective of serotype, with Rib and Alp 1 being the most common [12]. Naturally acquired antibodies against the Alp proteins exist in most individuals, and antibodies against full-length Alpha C and Rib proteins correlate inversely with risk of invasive GBS disease in the neonate [13]. Furthermore, antibodies to full-length Alp3 confers protection in animal models of invasive GBS disease [14].

Members of the Alp family are anchored to the cell wall at the C-terminus and extend a functionally active *N*-terminal domain out through the polysaccharide capsule. The *N*-terminal domain contains glycosaminoglycan- and integrin-binding sites, and is involved in invasion of epithelial cells by GBS [15], [16], and deletion of the *N*-terminal domain attenuates virulence [17], indicating a role in the pathogenicity of GBS. Likewise, antibodies targeting the *N*-terminal domains of Rib and Alpha C has been demonstrated to confer protection in animal invasive GBS disease models [13], [18]. Alp 2 and 3 share the same *N*-terminal domain, meaning that only five different Alp *N*-terminal domains exist. Extensive sequence homology exists between these five domains [9], [11], and the sequences are surprisingly well preserved between clinical isolates with only few and mostly conservative single amino acid point mutations identified, primarily in the *N*-terminal domains of Rib and Alp 1[12](Beall, B - personal communication). Furthermore, Alp 4 is extremely rare and of the 6340 invasive GBS isolates collected and sequenced by the CDC during the period of 2015–2017, no Alp 4 expression was detected whereas the remaining four Alp *N*-terminal domains (Rib-N, AlphaC-N, Alp1-N, Alp2/3- N) were detected in 99.3 % of these invasive isolates [12]. Together this indicates that only four Alp *N*-terminal domains are relevant for invasive GBS disease.

Low levels of naturally occurring antibodies against multiple GBS surface antigens have been associated with an increased risk of invasive GBS disease [13], [19]. Estimating a threshold of naturally occurring antibodies that correlate with a reduced risk of disease could be used as surrogate endpoints for licensure of a protein-based GBS vaccine. In this exploratory study, we investigated the association between maternal derived infant serum IgG against the RibN and Alp1N domains and risk of invasive GBS disease, and estimated antibody thresholds that correlate to protection against disease.

## 2 Methods

We undertook an analysis of stored samples from a case-control study undertaken at the Chris Hani Baragwanath Academic Hospital, Charlotte Maxeke Johannesburg Academic Hospital and the Rahima Moosa Mother and Child Hospital in Johannesburg from 2012 to 2014 [20]. The “risk-based” rather than “universal screening” strategy for use of intrapartum antibiotic prophylaxis is standard of care in this study setting. Neonates or infants<3 months of age with signs and symptoms of sepsis and/ or meningitis routinely have blood and cerebrospinal (CSF) fluid cultures.

The mother was approached for consent and enrolment of her child and herself within 48 h of the culture result becoming available. As part of the consenting, mothers signed consent for storage and usage of samples for further testing of antibodies to other GBS antigens subject to approval by the HREC. Controls (mothers of well infants) were selected from hospital registries and postnatal wards. Bloods were taken from the mother (5 mL) and baby (1–2 mL) or cord (5 mL) for serum IgG against targeted GBS epitopes. The women had a rectal and lower vaginal swab which was sent for GBS culture and serotyping as described [21].

GBS isolates from colonized women and invasive disease isolates from the infants were tested for the presence of *N*-terminal domains of Rib (RibN) and Alp1 (Alp1N) IgG using PCR at the State Serum Institute, Denmark, as described [22]. Briefly, Alp protein typing on the GBS isolates from cases and colonized controls was performed by PCR using primers that target the genomic regions for the *N*-terminal domains of RibN, AlphaCN, Alp1N, and Alp 2/3N.

### 2.1 Cloning and production of recombinant RibN amd Alp1N proteins

Recombinant Rib and Alp1 *N*-terminal domains were produced and purified by Bioneer A/S, Denmark, at the commission of MinervaX (Denmark) as recently described [8].

### 2.2 Quantitation of RibN and Alp1N specific serum IgG

For quantitation of RibN and Alp1N specific serum IgG, a commercial human immunoglobulin for subcutaneous administration (Subcuvia, 160 mg/mL, Baxalta, Sweden) (SCIG) was used as a standard. The concentration of RibN and Alp1N specific IgG in the SCIG preparation was initially determined based on the method of equivalence of absorbance between a reference ELISA and respective anti-AlpN ELISA [23], [24], as recently described [7], [8].

Using the SCIG preparation as a calibrated assay standard, the concentrations of RibN and AlpN specific IgG in maternal and neonatal serum samples were determined by ELISA, as recently described [7], [8]. Briefly, 96-well microtiter plates were coated with 100 µl of RibN or Alp1N all at 0.5 µg/mL in PBS overnight at 4 °C. After washing with PBS-0.05 % Tween 20 (wash buffer), serum from the study subjects was serially diluted in PBS-2 % BSA-0.05 % Tween 20 (sample buffer) and added to the wells (100 µl/well). Calibrated SCIG was serially diluted in sample buffer and added to separate rows of wells on each plate (100 µl/well) as a standard with known concentration of antibodies specific to each AlpN protein. After two hour incubation at room temperature, the plate was washed and then incubated with horseradish peroxidase (HRP)-conjugated goat anti-human IgG (γ-chain) F (ab’)_2_-HRP (Sigma A2290) for one hour at room temperature. After washing, HRP was detected using tetrametylbenzidine (TMB; Sigma T8768)-hydrogen peroxide (100 µl/well). Colour reaction was stopped with 50 µl of 1 M sulfuric acid/well and the absorbance was read in SpectroStar photometer at 450 nm wavelength. Raw data were processed using 4PL fit function in Prism 7 for macOS (GraphPad). The working range of each assay was determined by repeated analyses of serially diluted SCIG standard (assayed in quadruplicates at each occasion), performed at different days and by at least two separate operators (in total four independent assays for each coating antigen). The working range was defined as the concentration interval in which the back calculated concentrations deviated ≤ 20 % from the theoretical value and the inter-assay coefficient of variation (CV) in the same time was ≤ 22 % (Supplementary Fig. 1). By this approach, the lower and upper level of quantification (LLOQ and ULOQ, respectively) for RibN IgG in the assay were set to 0.09 ng/mL and 12 ng/mL, respectively (i.e. the concentration measured in the wells). By analogy, the LLOQ and ULOQ for Alp1N IgG in the assay were determined to be 0.18 ng/mL and 24 ng/mL, respectively. Concentrations of antibodies in serum samples were then computed for all sample dilutions with absorbance values within the working range of the calibrated SCIG standard and results are given in μg IgG per mL of serum. The starting serum dilution factor in the assay was 1:25 and taking this into account, the LLOQ in the serum samples were 2 ng/mL and 4 ng/mL for RibN and Alp1N IgG respectively. Serum samples containing less than these values, were assigned 1 ng/mL and 2 ng/mL for RibN and Alp1N IgG, respectively (0.5 × LLOQ).

### 2.3 Statistical analysis

Demographic and clinical characteristics for invasive GBS cases and colonized controls were compared using the Chi-square, Fischer’s exact or Mann-Whitney test as indicated. Maternal and infant serum IgG geometric mean concentrations (GMC) were compared between cases and controls using the student *t*-test. A Bayesian model was used to calculate the probability that an infant would develop EOD or LOD given a GBS homotypic IgG concentration greater than or equal to *c*, over a grid of values for *c*. Details of these methods have previously been described [21]. Data were analyzed using STATA version 13.1 (College Station, Texas, USA), R version 2.15 (Vienna, Austria) and JAGS.

Informed consent was obtained from mothers of infants. The study was approved by the University of Witwatersrand Human Research Ethics Committee (HREC number: M150489, M120963).

## 3 Results

A total of 85 paired infant/maternal serum samples from cases of invasive GBS disease were available. PCR based typing of corresponding invasive GBS isolates showed that 46 (54.1 %) isolates expressed the Rib protein gene, 24 (28.2 %) Alp1, 12 (14.1 %) AlphaC and 3 (3.6 %) Alp2/3. Due to the low number of cases with AlphaC and Alp2/3 expressing strains, the analyses focused on Rib and Alp1 coding strains. The control group was constituted of women colonized with GBS strains expressing Rib (n = 46) and Alp1 (n = 36) proteins, whose children did not develop invasive GBS disease. Maternal and infant clinical characteristics were similar between the cases and controls, except for mothers of Alp1 cases more likely to have had prolonged rupture of membranes (≥18 h) (p = 0.030) (Supplementary Table 1). Most Rib expressing cases (83 %) and controls (61 %) were serotype III, and 92 % for cases and controls that expressed Alp1 were serotype Ia (Supplementary Table 2).

Infant RibN IgG GMC (μg/mL) were lower in cases (0.01; 95 %CI: 0.01–0.02; n = 46) than controls (0.04; 95 %CI: 0.03–0.06; n = 44; p < 0.001) (Table 1). No significant difference was found between maternal RibN IgG GMC in cases compared to controls. Infant Alp1N IgG GMC were also lower in cases (0.02; 95 %CI: 0.01–0.03; n = 24) than controls (0.045; 95 %CI: 0.04–0.07; n = 36; p < 0.001), albeit not differing between the mothers of cases and controls (Table 1).

**Table 1:** Geometric mean concentrations (μg/mL) between cases and control for maternal and **I**nfant IgG.

Protein	Variable	Case	Control	p-value^a^	log-rank p-value^b^
RibN	Maternal IgG	0.04 (0.03,0.07) n = 46	0.06 (0.04,0.08) n = 46	0.335	0.773
**RibN**	**Infant IgG**	**0.01 (0.01,0.02) n = 46**	**0.04 (0.03,0.06) n = 44**	**<0.001**	**0.007**
Alp1N	Maternal IgG	0.08 (0.05,0.13) n = 24	0.11 (0.08,0.16) n = 36	0.212	0.503
**Alp1N**	**Infant IgG**	**0.02 (0.01,0.03) n = 24**	**0.05 (0.04,0.07) n = 36**	**<0.001**	**0.001**

a p-value using *t*-test to compare the mean of the log antibody concentrations between cases and controls.

b p-value to compare the reverse cumulative curves for antibody concentrations between cases and controls.

**Fig. 1:**
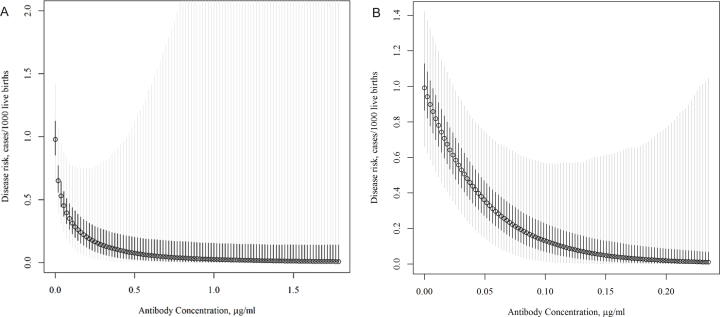
Probability of invasive group B streptococcal disease risk to RibN and AlpN type disease at varying infant serum IgG antibody concentrations using a Bayesian model. *Footnote*: The circles represent the posterior mode (i.e., the most likely value). The lighter, shaded, vertical bands indicate the 95% credible intervals, while the darker bands represent the 50% credible intervals.

**Fig. 2:**
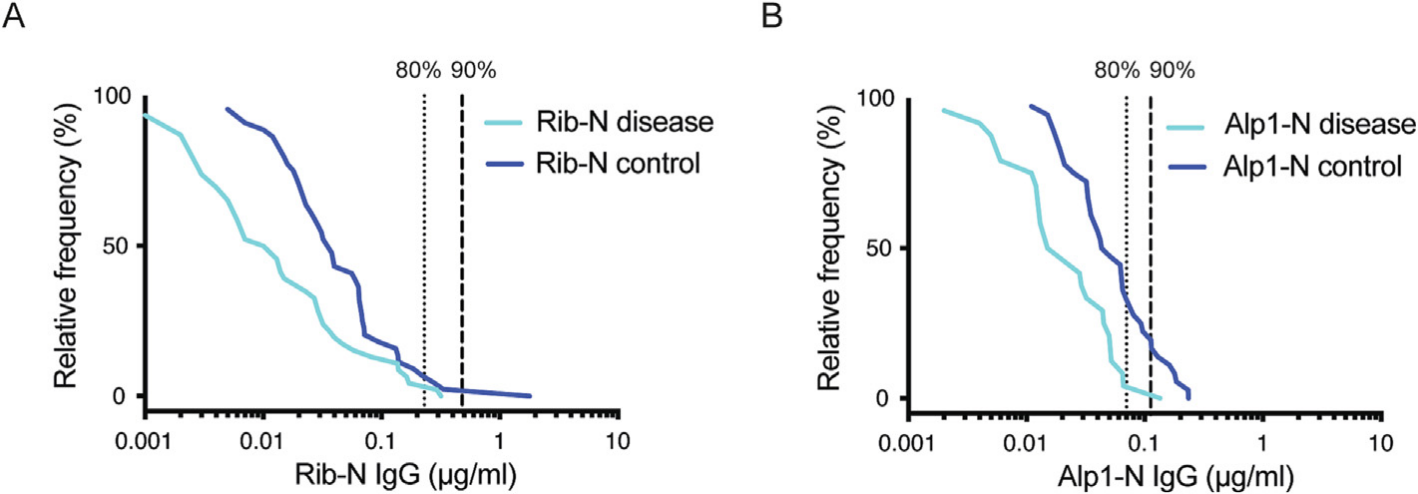
Cumulative distribution of RibN and Alp1N IgG antibody levels respectively in cases and controls. *Footnote*: Vertical dotted lines indicate the IgG level predicting 80 % disease reduction, and striped line indicate the IgG level predicting 90 % disease reduction as estimated by Bayesian modelling.

Using Bayesian analysis, an infant serum IgG threshold ≥ 0.428 µg/mL (95 % credible interval: 0.0048–1.0312) and ≥ 0.112 µg/mL (95 % credible interval: 0.0095, 0.5475) was associated with 90 % risk reduction of Rib and Alp1 expressing isolates, respectively (Fig. 1A & B). Plotting the cumulative distribution of RibN and Alp1N IgG levels for the case and control groups also showed a clear separation of the two curves, with IgG distribution for cases being lower than controls (Fig. 2A & B).

Stratified by EOD and LOD, maternal and infant RibN and Alp1N IgG GMC were lower in cases with LOD than controls (Supplementary Table 3). Amongst infants with LOD, a serum IgG threshold ≥ 0.125 and ≥ 0.046 µg/mL was associated with 90 % risk reduction of Rib and Alp1 expressing isolates (Supplementary Table 3 and Supplementary Fig. 2a-h).

## 4 Discussion

This study demonstrates an association between low infant serum IgG against the *N*-terminal domains of Rib and Alp1, members of the Alp protein family of GBS surface proteins, and increased risk of invasive GBS disease. Infant natural RibN and Alp1N IgG levels above 0.428 µg/mL and 0.112 µg/mL was associated with a 90 % risk reduction of invasive disease from Rib and Alp1 expressing GBS isolates respectively. A GBS vaccine containing the four highly conserved AlpN domains (RibN, AlphaCN, Alp1N and Alp2/3N) covering 99.3 % of clinical isolates is currently under clinical development. The presented threshold of RibN and Alp1N infant serum IgG and risk reduction of invasive GBS disease represent a preliminary guidance for the IgG levels to be targeted for transplacental transfer to the infant when vaccinating their mothers.

Notably, past seroepidemiogical studies on antibody associated with risk reduction of invasive GBS disease has mainly focused on serotype specific capsular antibody polysaccharide capsule rather than protein epitopes [19]. Multiple antibody specificities may contribute to natural protection against invasive GBS disease; hence there is a need to broadly characterise the association of protein and capsular epitope specific IgG and risk reduction of infant invasive GBS diseases. A hexavalent GBS polysaccharide protein conjugate vaccine with well-conserved protein epitopes, either as conjugate or add on, could theoretically enhance protection against invasive GBS disease.

Our findings of low levels of naturally occurring antibodies and risk of invasive disease have been corroborated by others – low full-length Rib and AlphaC proteins antibody levels were associated with an increased risk of invasive neonatal GBS disease [13], and investigational vaccines comprising RibN and AlphaCN, full-length AlphaC or Alp3 were found to protect against invasive GBS disease in animal models [14], [18], [25].

Maternal antibody levels were also lower in cases than controls albeit not significant. Other studies investigating select GBS surface proteins (such as fibrinogen-binding protein A and the putative GBS-2106 protein) have also demonstrated an inverse association between maternal antibody levels and risk of invasive GBS disease [21](Madhi - personal communication). The European DEVANI study showed an association between pilus-island 1 and 2a proteins and invasive early-onset disease; however, no association was found in a South African cohort [21]. The transplacental transfer of protein epitopes, which are predominantly IgG1, are reported to be more efficient than polysaccharide epitopes which are mainly IgG2 [26]. Therefore, in setting where serum from infants cannot be obtained, measuring maternal antibody levels against protein epitopes in seroepidemiological studies may provide an adequate reflection of the maternally derived infant IgG levels.

Our study had some limitations. AlphaC and Alp2/3 expressing isolates were insufficient for statistical modelling, hence no correlates of protection could be developed IgG levels against the AlphaCN and Alp2/3N domains. A further limitation is that we did not measure opsonophagocytic killing titres. Alp family antibodies induce opsonophagocytic killing of GBS isolates in vitro [8]. Also, this was a single country study and further studies and in other settings are warranted.

In conclusion, RibN and Alp1N infant serum IgG of greater or equal to 0.428 µg/mL and 0.112 µg/mL respectively, were associated with 90 % risk reduction of invasive GBS disease. Such data could contribute to the pathway of licensure of a maternal GBS vaccine that targets these epitopes based on safety and immunological correlates suggestive of protection against invasive GBS disease.

## Declaration of Competing Interest

The authors declare the following financial interests/personal relationships which may be considered as potential competing interests: Financial disclosure/support: The study was funded the Bill and Melinda Gates Foundation (OPP1134339) and by MinervaX ApS. SAM institution received funding from National Research Foundation/Department of Science and Technology: South African Research Chair Initiative in Vaccine Preventable Diseases and South African Medical Research Council of South Africa. The funders had no role in study design, data collection and analysis, decision to publish, or preparation of the manuscript. Conflict of interest: PBF and BJL are employees of MinervaX ApS developing a Group B Streptococcal vaccine based on the N-terminal domains of the Alpha-protein family of GBS surface proteins, incl. RibN and Alp1N.

## Data Availability

Data will be made available on request.
