# Supplementary material for: Association of infant Rib and Alp1 surface protein N-terminal domain immunoglobulin G and invasive Group B Streptococcal disease in young infants
Source: Vaccine. 2023 Mar 3;41(10):1679–83. doi: 10.1016/j.vaccine.2023.01.071 (PMC9996286; doi:10.1016/j.vaccine.2023.01.071)
Supplement: Supplementary data 1 [file mmc1.docx]

Supplementary Table 1: Demographic and clinical characteristics between cases and controls

|  |  | **Rib** | | **Alp1** | |
| --- | --- | --- | --- | --- | --- |
|  |  | Case; n=46 (EOD^a^=16, LOD^b^=30) | Rib colonised control; n=46 | Case; n=24  (EOD=13, LOD=11) | Alp1 colonised control; n=36 |
| **Maternal age (years)** | <25 | 16 (34.8%) | 22 (47.8%) | 14 (58.3%) | 16 (44.4%) |
|  | 25-<35 | 27 (58.7%) | 20 (43.5%) | 9 (37.5%) | 17 (47.2%) |
|  | >=35 | 3 (6.5%) | 4 (8.7%) | 1 (4.2%) | 3 (8.3%) |
| **Gestational age (weeks)** | <34 | 8 (17.4%) | 2 (4.3%) | 2 (8.3%) | 4 (11.1%) |
|  | 34-<37 | 4 (8.7%) | 10 (21.7%) | 5 (20.8%) | 6 (16.7%) |
|  | >=37 | 34 (73.9%) | 34 (73.9%) | 17 (70.8%) | 26 (72.2%) |
| **Parity** | 0 | 11 (23.9%) | 19 (41.3%) | 11 (45.8%) | 9 (25%) |
|  | 1 | 21 (45.7%) | 14 (30.4%) | 7 (29.2%) | 18 (50%) |
|  | 2 | 12 (26.1%) | 9 (19.6%) | 4 (16.7%) | 9 (25%) |
|  | 3 | 1 (2.2%) | 3 (6.5%) | 2 (8.3%) | 0 (0%) |
|  | 4 | 1 (2.2%) | 0 (0%) |  |  |
|  | 5 | 0 (0%) | 1 (2.2%) |  |  |
| **Gravida** | 1 | 10 (21.7%) | 19 (41.3%) | 9 (37.5%) | 8 (22.2%) |
|  | 2 | 19 (41.3%) | 12 (26.1%) | 8 (33.3%) | 19 (52.8%) |
|  | 3 | 14 (30.4%) | 9 (19.6%) | 4 (16.7%) | 8 (22.2%) |
|  | 4 | 1 (2.2%) | 4 (8.7%) | 3 (12.5%) | 1 (2.8%) |
|  | 5 | 1 (2.2%) | 2 (4.3%) |  |  |
|  | 7 | 1 (2.2%) | 0 (0%) |  |  |
| **Maternal HIV** | Negative | 23 (50%) | 26 (56.5%) | 15 (62.5%) | 21 (58.3%) |
|  | Positive | 23 (50%) | 20 (43.5%) | 9 (37.5%) | 15 (41.7%) |
| **ROM^c^** | >=18h | 4 (12.5%) | 4 (9.1%) | 5 (22.7%) | 1 (2.9%) |
|  | <18h | 28 (87.5%) | 40 (90.9%) | 17 (77.3%) | 33 (97.1%) |

^a^Early-onset disease; ^b^Early-onset disease; ^c^Rupture of membranes

Supplementary Table 2: Capsular polysaccharides serotypes of Rib and Alp1 isolates from

neonatal invasive disease (Cases) and colonized mothers (Controls).

|  | **Rib strains** | | **Alp1 strains** | |
| --- | --- | --- | --- | --- |
|  | Cases | Controls^a^ | Cases | Controls^b^ |
|  | (n=46) | (n=46) | (n=24) | (n=36) |
| Ia | 3 (7%) | 7 (15%) | 22 (92%) | 33 (92%) |
| Ib | 0 (0%) | 0 (0%) | 0 (0%) | 2 (6%) |
| II | 2 (4%) | 8 (17%) | 0 (0%) | 1 (3%) |
| III | 38 (83%) | 28 (61%) | 0 (0%) | 0 (0%) |
| V | 3 (7%) | 4 (9%) | 2 (8%) | 1 (3%) |
|  |  |  |  |  |

1. Sum > 100% due to detection of two CPS types in the same subjects

(n=2)

1. Sum > 100% due to detection of two CPS types in one subject

Supplementary Table 3: Geometric mean concentrations (μg/mL) between cases and control for maternal and Infant IgG stratified by early- and late-onset disease

| Protein | Measurement | Disease | Case | Control | p-value | non-parametric  p-value | log-rank p-value | Estiamted 90% threshold of protection* |
| --- | --- | --- | --- | --- | --- | --- | --- | --- |
| RIB-N | Maternal IgG | EOD | 0.06 (0.03,0.11) n=16 | 0.05 (0.04,0.07) n=22 | 0.528 | 0.734 | 0.375 | - |
| RIB-N | Maternal IgG | LOD | 0.04 (0.02,0.06) n=30 | 0.06 (0.04,0.1) n=24 | 0.143 | 0.156 | 0.364 | 0.840 |
| RIB-N | Infant IgG | EOD | 0.03 (0.01,0.06) n=16 | 0.06 (0.04,0.09) n=20 | 0.086 | 0.091 | 0.315 | - |
| RIB-N | Infant IgG | LOD | 0.01 (0,0.01) n=30 | 0.03 (0.02,0.05) n=24 | 0.001 | 0.001 | 0.013 | 0.125 |
| ALP1-N | Maternal IgG | EOD | 0.08 (0.04,0.18) n=13 | 0.09 (0.05,0.16) n=14 | 0.860 | 0.752 | 0.568 | - |
| ALP1-N | Maternal IgG | LOD | 0.03 (0.02,0.05) n=13 | 0.08 (0.05,0.12) n=14 | 0.004 | 0.012 | 0.01 | - |
| ALP1-N | Infant IgG | EOD | 0.07 (0.04,0.15) n=11 | 0.13 (0.08,0.21) n=22 | 0.160 | 0.147 | 0.419 | 0.115 |
| ALP1-N | Infant IgG | LOD | 0.01 (0.01,0.02) n=11 | 0.04 (0.03,0.05) n=22 | 0.004 | 0.004 | 0.002 | 0.046 |

*Bayesian analysis


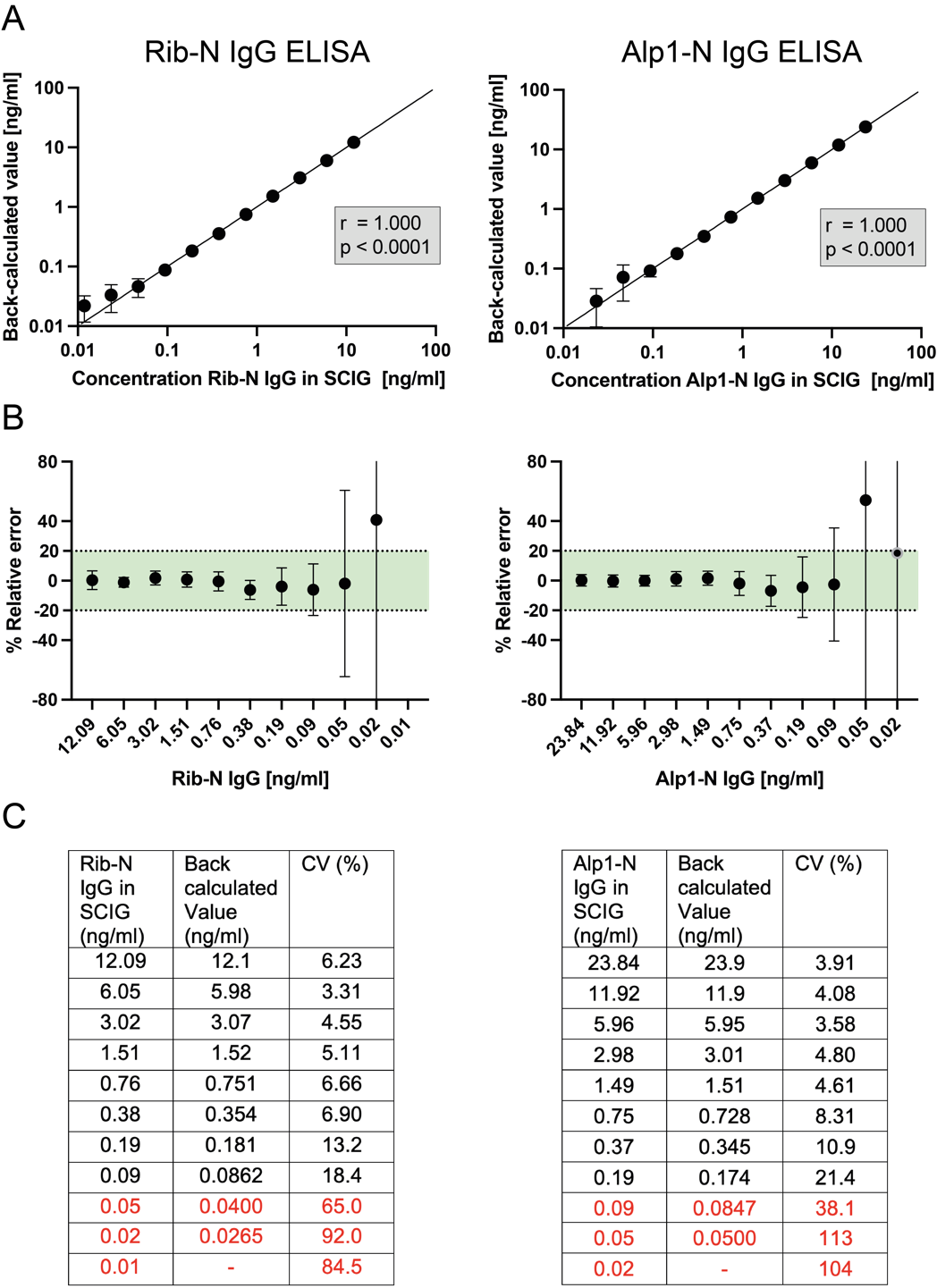


**Supplementary Figure 1.** **Working range of the RibN and Alp1N IgG ELISAs with SCIG (Baxalta) as standard**. The concentrations of RibN and Alp1N specific IgG in the SCIG preparation were initially determined as described in material and methods (1.27 µg/ml and 2.19 µg/ml of RibN and Alp1N specific IgG, respectively). Serially diluted SCIG was then assayed in quadruplicates at four different days and by at least two separate operators. Standard curves were generated separately for each serial dilution, using the 4PL fit function in Prism 7 for macOS (GraphPad). (**A**) Concentrations back-calculated from individual absorbance values, using the 4PL standard curve derived from the SCIG entire dilution series, plotted against the expected concentrations of RibN and Alp1N IgG, respectively. Mean values ± 95% CI are shown (n=16). (**B**) Errors of back calculated values relative to the expected concentrations of RibN and Alp1N IgG, respectively. Mean relative errors ± SD are shown (n=16). Green area defines relative errors < 20%. (**C**) Mean back-calculated concentrations and coefficient of variations (CV) achieved for all individual measurements and each individual dilution of the SCIG preparation (n=16). Red values correspond to CV > 22% and were defined as outside the working range of the assay.


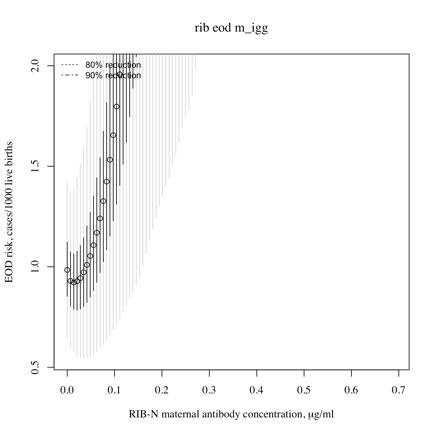

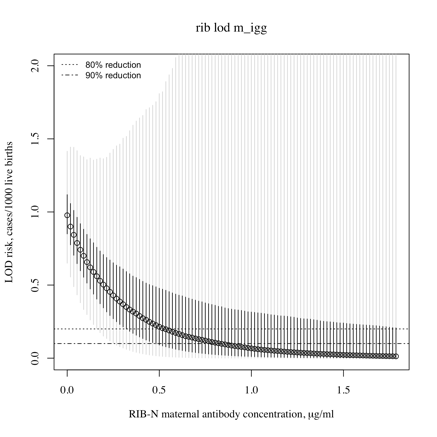


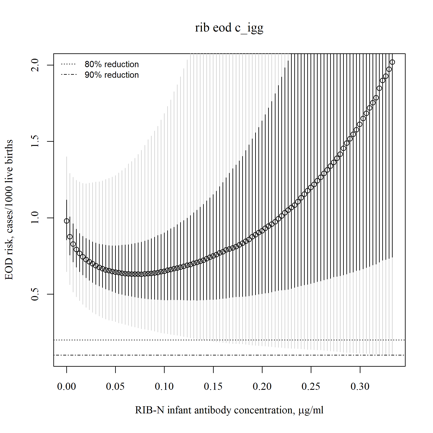

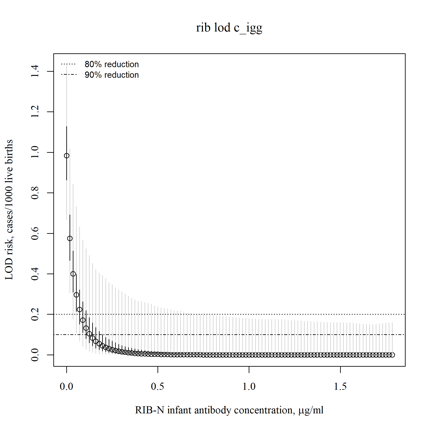


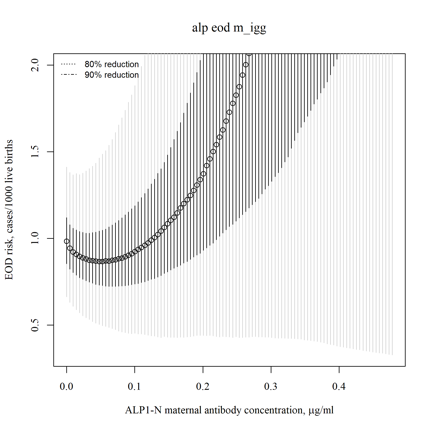

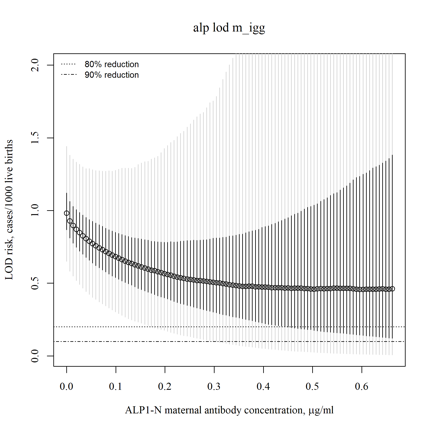


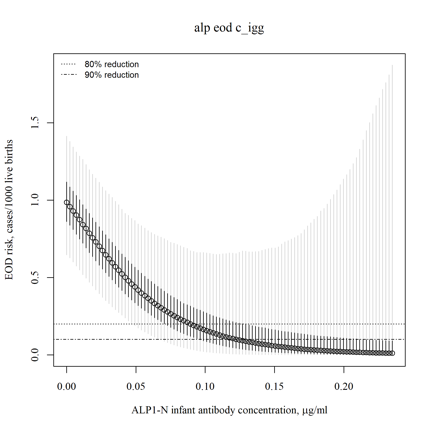

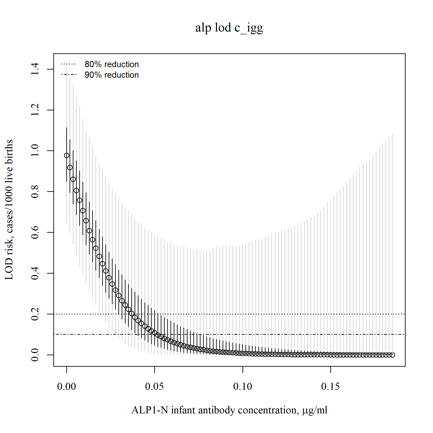


**Supplementary Figures 2 (a-h): Probability of invasive group B streptococcal early and late-onset disease risk to RibN and AlpN type disease at varying maternal and infant serum IgG antibody concentrations using a Bayesian model**. The circles represent the posterior mode (ie, the most likely value). The lighter, shaded, vertical bands indicate the 95% credible intervals, while the darker bands represent the 50% credible intervals.
